# Application of Dendrimers for Treating Parasitic Diseases

**DOI:** 10.3390/pharmaceutics13030343

**Published:** 2021-03-05

**Authors:** Veronica Folliero, Carla Zannella, Annalisa Chianese, Debora Stelitano, Annalisa Ambrosino, Anna De Filippis, Marilena Galdiero, Gianluigi Franci, Massimiliano Galdiero

**Affiliations:** 1 Department of Experimental Medicine, University of Campania “Luigi Vanvitelli”, 80138 Naples, Italy; veronica.folliero@unicampania.it (V.F.); carla.zannella@unicampania.it (C.Z.); annalisa.chianese@unicampania.it (A.C.); debora.stelitano@unicampania.it (D.S.); annalisa.ambrosino@unicampania.it (A.A.); marilena.galdiero@unicampania.it (M.G.); 2 Department of Pharmacy, University of Naples “Federico II”, 80131 Naples, Italy; anna.defilippis@unina.it; 3 Department of Medicine, Surgery and Dentistry “Scuola Medica Salernitana”, University of Salerno, 84081 Baronissi, Italy; gfranci@unisa.it

**Keywords:** dendrimer, nanostructure, parasite, drug delivery

## Abstract

Despite advances in medical knowledge, parasitic diseases remain a significant global health burden and their pharmacological treatment is often hampered by drug toxicity. Therefore, drug delivery systems may provide useful advantages when used in combination with conventional therapeutic compounds. Dendrimers are three-dimensional polymeric structures, characterized by a central core, branches and terminal functional groups. These nanostructures are known for their defined structure, great water solubility, biocompatibility and high encapsulation ability against a wide range of molecules. Furthermore, the high ratio between terminal groups and molecular volume render them a hopeful vector for drug delivery. These nanostructures offer several advantages compared to conventional drugs for the treatment of parasitic infection. Dendrimers deliver drugs to target sites with reduced dosage, solving side effects that occur with accepted marketed drugs. In recent years, extensive progress has been made towards the use of dendrimers for therapeutic, prophylactic and diagnostic purposes for the management of parasitic infections. The present review highlights the potential of several dendrimers in the management of parasitic diseases.

## 1. Introduction

Diseases caused by parasites are still a serious problem for human health despite the widespread advancement in medical technology. These diseases, although more present in developing countries, represent a weighted risk to health in many developed areas, in fact several factors influence the presence of parasitosis at a more global level such as: a significant increase in international tourism, policies of immigration, the international trade in food products, the public’s lack of awareness and knowledge of parasites, the growing number of immunosuppressed and aging people. Therefore, despite the continuous advances in scientific and medical knowledge, parasitic diseases are still present today with high morbidity and mortality in vast geographical areas.

Parasitic diseases are infections caused or transmitted by parasites, eukaryotic organisms that live inside or on a host, in which they complete part or all of their life cycle [1]. Most parasites involved in human parasitic diseases belong to two categories: protozoa and helminths [2]. The most important protozoan parasites include the genus Plasmodium, Entamoeba, Acanthamoeba, Leishmania, Trypanosoma and Toxoplasma [3]. Instead, the helminths of major medical importance belong to the genus of Ascaris, Schistosoma and Tenia [4].

Transmission of protozoa occurs through the ingestion of contaminated water or food, person-to-person contact or bites from arthropod vectors. These diseases have a strong impact on public health and represent a major cause of morbidity and mortality worldwide. In addition, parasitic infections lead to serious socio-economic losses in the population, especially in developing countries. The World Health Organization (WHO) has estimated that over 3 billion people are affected by one or more parasitic infections, globally [5]. Eleven parasitic infections have been identified by the WHO as underrated tropical diseases that threaten the health of millions of individuals living in disadvantaged social conditions [6,7].

Parasitic infections are not limited to tropical regions of the world, in fact the Centers for Disease Control and Prevention (CDC) have identified five parasitic infections with a significant public health impact for people living in the United States. The outcome of parasitic disease is often related to the host immune status [8]. Humans protect themselves from parasites using a variety of defenses, including nonspecific immunity, innate mechanisms, and adaptive immune mechanisms. In general, helminths induce a host response of type 2 helper T lymphocytes (Th2) with production of immunoglobulin (Ig) E, eosinophilia and mastocytosis, while protozoa induces a humoral and/or cellular immune response not associated with eosinophilia. Infants, the elderly and immunocompromised individuals are more susceptible to parasitic infections [9].

Despite the latest progress, there are currently no vaccines against the main human parasitic infections such as malaria, amoebiasis, leishmaniasis and schistosomiasis [10]. According to Ada and colleagues, the principal reasons for this include: (i) it is cumbersome to grow parasites in large enough amounts to produce vaccines used to immunize all people at risk; (ii) parasites are characterized by different morphological forms during their life cycle; an effective vaccine must protect the host from all life cycle parasite forms to generate an appropriate protective immune response; (iii) parasites have evolved several strategies to evade the immune responses; (iv) parasitic antigens involved in the immune response are difficult to detect [11]. Although vaccination is the most advantageous method for the control and management of parasitic infections, chemotherapy is actually the major available option [12]; however, therapeutic options are limited due to problems related to efficiency, side effects, increases in drug resistance and high costs. In order to solve these problems, a wide variety of drugs has been tested in vivo and in vitro against parasites [13]. Amphotericin B (AmB) represents a first-line therapy for the treatment of several parasitic diseases, but its use has been hindered by several limitations such as toxicity and emergence of drug resistance that can lead to treatment failure. Within different parasites, several resistance mechanisms can rapidly emerge. The main adverse effects of AmB include alteration in renal function, hematologic toxicity, liver damage, hypoxia, fever and nausea. At present, safe and effective anti-parasitic drugs are lacking [14]. Considering the high impact of parasitic infections on public health, the limited availability of effective and safe drugs and the rapid spread of drug resistance, it is necessary to develop new drugs that can effectively treat parasitic diseases. In recent years, the development of novel drug delivery systems as potential therapeutic carriers has provided us with a large variety of biotechnological applications [15]. Delivery systems, involved in the treatment of infectious diseases, include nanoparticles, micelles, nanogels, dendrimers etc. [16,17] Dendrimers represent highly branched polymers with a functionalizable surface [18], providing several advantages compared to conventional drugs. For instance, dendrimers deliver drugs to target sites with a reduced dosage to decline the adverse effects that occur with conventional therapies. In addition, they solve problems encountered with traditional drugs such as low specificity, fast elimination, and low bioavailability [19]. These characteristics lead to an increase in the effectiveness of the treatment and a reduction in toxicity and adverse reactions caused by current drugs in use [20]. The current review highlights the biological efficacy of several dendrimers in the management of parasitic diseases.

## 2. Methodology

**Figure 1:**
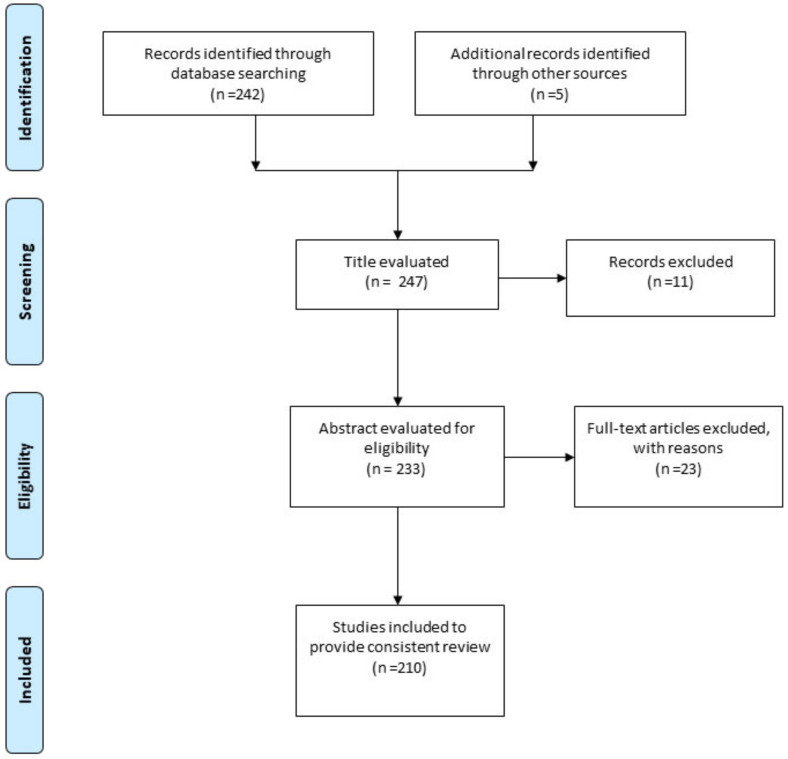
PRISMA flow diagram, showing the process of study selection.

The present study consists of an up-to-date review of literature on application of dendrimers to treat parasitic diseases. A number of electronic databases were used for the literature search including Scopus, Cochrane library, Web of Science, Google scholar and PubMed, using the keywords of “parasite”, “parasitic infections”, “parasitic disease”, “drug-delivery”, “dendrimers”, “polymers”. The criteria of selecting articles were as follows: a) only studies reported in English language; b) studies related to dendrimers for treatment of parasitic disease; c) in vitro/in vivo studies and clinical trials. The results returned 213 papers published up to 2020, which were summarized and critically discussed to provide a consistent review. Some books and official WebPages (World Health organization) were also used for citing specific data related to the scope of the present study. Figure 1 illustrates the Preferred Reporting Items for Systematic Reviews and Meta-Analysis (PRISMA ) flow diagram for study selection.

## 3. Dendrimer: A Tree-Like Polymer with Many Branches of Applications

**Figure 2:**
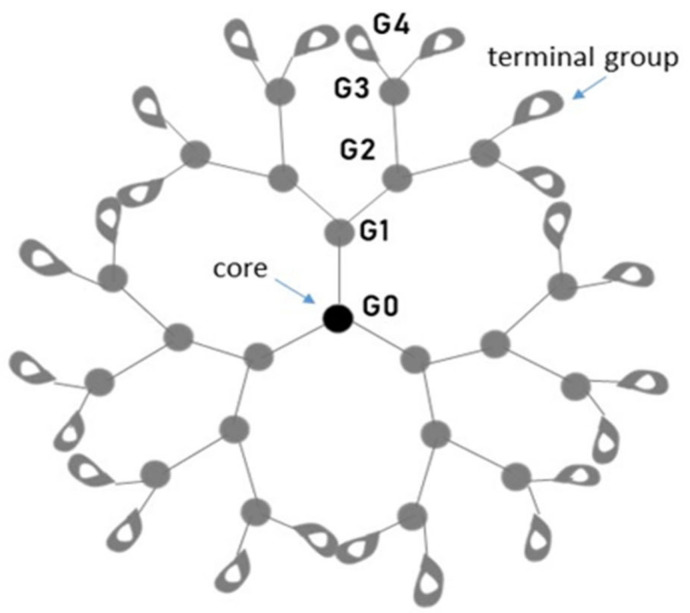
Schematic representation of dendrimer. Core, branches and terminal groups are present from the inside to the outside of the macromolecule.

Polymer science represents a multifaceted area with different fields of application and is therefore highly intriguing in modern research. Dendrimers have emerged as one of the brightest and most explored polymers for different scientific fields, including medicine where they may act as wide platforms to transfer therapeutic and diagnostic agents. The Greek origins of the dendrimer terms (dendros, meaning “tree”, and meros, “part”) clearly reflect their nature: three-dimensional (3D) structures of nanometric size range (1–100 nm) with tree-like branched arrangements. Dendrimers were firstly discovered by Vogtle in 1978 and, subsequently, by Tomalia and Newkome independently in 1985 [21,22,23]. In the literature, dendrimers were called in different manners, “cascade molecules”, “arborols”, “dendritic molecules”, although they share common and peculiar characteristics. Dendrimers are branched polymeric macromolecules with well-defined dimensions structured in three domains: (i) a central core; (ii) branches, winding from the core; and (iii) terminal functional groups that enclose the dendritic structure [24] (Figure 2).

The dendrimer core represents its innermost part, consisting of one or a group of atoms closely associated to the branches, which extend over a wide-range. The branches are arranged to form a concentric molecule: several units are repeated, producing progressive spherical layers termed “generations” [25,26]. Accordingly, the dendrimer name refers to the number of generations used to build it and, sometimes, the core of the molecule is indicated as “zero generation” (G0). For example, polyamidoamine (PAMAM) dendrimers are also called G5-PAMAM or “fifth generation” dendrimers as a result of five branching points unfolding towards the surface. In the outermost part of the dendritic structure, many functional groups may be present. They are known as “terminal” or “surface groups” and, together with the branches, define the dendrimer’s physicochemical and biological features [20]. The modification of terminal groups alters cellular interactions and distributions of dendrimers in the biological system [27,28]. In the complete molecule, distinct and flexible “void spaces” are created: they represent the key element of dendrimers acting as “box” structures for guest molecules [29]. Many chemical procedures are used for the production of dendrimers, which drive the specific characteristics that the final product will exert in terms of solubility, degradability, but above all, biological activity. The most common dendrimers used in biomedical applications are made up of polyamidoamines (PAMAM) [30], polyamines [31], polyamides (polypeptides) [32], poly(aryl ethers) [33], polyesters [34,35], carbohydrates [36] and DNA [37,38]. By far the most prevalent scaffolding for dendrimers is PAMAM, easily available on the market and with the ability to offer a wide range of generations and surface features. Dendrimers are, therefore, one of the great successes within nanotechnology and in particular of medicinal chemistry. Their branched structure provides the essential characteristic to allow the application on their external surface of a large plethora of ligands that can be of enormous impact to interfere in a multivalent way with the adhesion capacity between microorganisms and their proteins with host tissues and cells in the early phases of infection. In many contexts, dendrimers can function directly as drugs but very often they can be used to transport a myriad of different pharmacologically active molecules. In such cases, drug interaction of the dendrimer or drug loading can be achieved in several ways: (1) encapsulation within the dendrimer; (2) electrostatic encapsulation; (3) covalent conjugation. Nanomedicine is therefore one of the most outstanding dendrimer applications. For instance, they have been widely used as nanocarriers for drug delivery [39,40,41,42,43,44], as diagnostic [45,46,47,48,49] and antimicrobial agents [50,51,52,53,54,55,56,57]. Advances in synthetic chemistry and characterization approaches have allowed the production of many different types of dendrimers with distinct functionalities along with their applications. In Table 1 we summarize the principal dendrimer types reporting, besides, products commercially available and their potential utilization.

## 4. Application of Dendrimers in Parasitic Infections

In recent years, the attention of the scientific community has focused on several applications of dendrimers. Owing to their unique physio-chemical properties, such as their uniform size, high level of branching, polyvalency, solubility in water and biocompatibility, dendrimers represent a valid carrier in drug delivery applications. Despite the huge literature describing dendrimer applications, an overview of their activity against protozoan and multicellular parasites is still limited [54].

The spread of noxious parasite diseases is also a consequence of the reduced development of novel therapeutic strategies together with the rapid diffusion of resistant species [78]. Moreover, due to the toxic effects often produced by many antiparasitic drugs, physicians are prone to prescribe antiparasitic drugs in low doses for prolonged therapeutic regimens [78,79,80,81], further increasing the spread of chemotherapy-resistant species. Dendrimers are attracting much interest because of their antimicrobial activity and their possible exploitation as drug carriers. This paragraph aims to summarize emerging efforts for the application of dendrimers for the prevention and treatment of parasitic infections. We have focused on five of the major parasitic diseases that afflict millions of people worldwide, specifically malaria, leishmaniasis, schistosomiasis, toxoplasmosis and acanthamebiasis. The failure to respond to the increasing demand for effective antiparasitic drugs made it imperative to explore new avenues; therefore, dendrimers seem to represent an excellent therapeutic alternative.

### 4.1. Malaria

Malaria is a parasitic infection caused by Apicomplexan protozoa, belonging to the Plasmodium genus [82]. More than 120 species are included in this genus, but only 5 infect humans: *Plasmodium ovale*, *Plasmodium malariae*, *Plasmodium vivax*, *Plasmodium falciparum* and *Plasmodium knowlesi*. *P. falciparum* and *P. vivax* represent the two main species implicated in malaria worldwide [83]. It has been estimated that around 219 million malaria cases occurred in 2017, which led to 435,000 deaths. People living in developing countries are the most affected, with around 90% of deaths in Africa [84]. Malaria is also common in Asia and Latin America [85]. Despite the increase in preventive and control measures, 3.4 billion people are still at risk of infection [86]. The disease is transmitted through the bite of an infected female mosquito (the vector) belonging to the genus Anopheles. Transmission can also occur through blood transfusion, organ transplants and the sharing of medical devices [87]. The parasites conduct a complex life cycle, consisting of a sexual stage within the vector and an asexual stage in the liver and bloodstream of the human host. During its biological cycle, the parasite assumes several morphological stages: sporozoite, merozoite, trophozoite and gametocyte [88]. The circumsporozoite protein of sporozoites binds the heparan sulfate proteoglycans (HSPGs) on the surface of hepatocytes, thus allowing their invasion. HSPGs of hepatocytes show a higher sulfation level compared to HSPGs of other organs, suggesting a selective targeting of sporozoites to hepatocytes [89]. *P. vivax* can produce a dormant stage within human liver tissue, in fact not all sporozoites are able to mature into schizonts, and some remain in a dormant stage as hypnozoites. This phase is clinically silent and represents the target of prophylactic or vaccine approaches, especially against *P. vivax* hypnozoites. Infected hepatocytes release merozoites into the bloodstream [90]. Erythrocyte binding-like and reticulocyte binding-like proteins are located on the merozoite surface and are involved in the invasion of red blood cells. In the red blood cells, merozoites replicate asexually, causing the lysis of the erythrocytes and the release of new merozoites into the bloodstream to start the process again. In erythrocytes, asexual reproduction determines the exponential increase in the number of parasites. The erythrocyte phase of the infection is responsible for clinical signs of malaria and represents the main target of chemotherapy. Pathogenesis of malaria disease is mainly associated with: (i) hemolysis, phagocytosis of parasitized erythrocytes and impaired erythropoiesis; and (ii) immune response unleashed by the release of parasites. Erythrocyte loss results in anemia, the severity of which is related to the level of the parasitemia [91]. Immune response determines the release of cytokines, reactive oxygen species and other cellular products involved in fever, sweating, weakness, weight loss, nausea, vomiting and other systemic clinical signs [92]. The typical symptom of malaria is fever. In different forms of malaria, fever is periodic, based on the duration of erythrocytic asexual cycle: 48 h for *P. vivax*, *P. falciparum* and *P. ovale* (“tertian malaria”); 72 h for *P. malariae* (“quartan malaria”). Fever, caused by *P. falciparum*, is usually irregular and does not exhibit a distinct periodicity [93]. The main malaria chemotherapy agents are artemisinin, primaquine, chloroquine, mefloquine and lumefantrine [94]. Artemisinin is a sesquiterpene lactone. It is a prodrug whose activation is iron-dependent. This causes an abundant release of free redox-active heme and ferrous iron whose presence is highly associated with parasitic damage. Activated artemisinin induces the formation of reactive oxygen species and the depolarization of the mitochondrial and cytoplasmic membranes of the parasite [95]. Primaquine is an 8-aminoquinoline and is active on all the hepatic phases of the parasite. Although the mechanism is unclear, the drug is thought to alter the parasite’s cellular respiration, generating free oxygen radicals [96]. Chloroquine is a 4-aminoquinoline and is a schizonticide drug. In the erythrocyte, the parasite digests the hemoglobin, forming amino acids and heme. Amino acids are used by the parasite as a source of nutrition, while the heme group is toxic. To this end, plasmodes convert heme into hemozoin, non-toxic to the parasites. Chloroquine prevents the formation of hemozoin, causing the death of the plasmodium [97]. Mefloquine is a quinoline derivative and its mechanism of action is not completely understood, though it may act as a protein synthesis inhibitor [98]. Lumefantrine is a racemic fluorene derivative, administered in combination with artemether to improve its effectiveness. This drug prevents the formation of hemozoin [99]. Unfortunately, most of these drugs have been proved to cause moderate to serious side effects such as stomach pain, diarrhea, vomiting and visual disturbances [100].

Recently, dendrimers have been studied for the management of antimalarial drugs. In a study of Bhadra et al., poly-L-lysine-based peptide dendrimers with a PEG amine core (5G) coated with chondroitin sulfate A (CSA) were used for controlled and extended release of chloroquine phosphate (CQ). CSA coating significantly increased the drug loading from 9 to 27 CQ molecules per dendrimer and significantly increased drug release from 2 to 3 times. Unlike CQ-loaded uncoated dendrimers, CSA-coated nanocarriers were more stable at room temperature (25 °C) and could be refrigerated (2–8 °C) for up to five weeks. Antimalarial drugs and peptide dendrimers exhibited a notable hemolytic toxicity due to their numerous cationic charges. The assays showed a significant reduction in the hemolytic toxicity of the CQ drug. There was a decrease of 12.4% in CQ-loaded uncoated nanocarriers, while a reduction of 34.1% in CQ-CSA coated dendrimers was recorded. Toxicity was assessed on mouse peritoneal macrophages. CQ cytotoxicity was reduced by the peptide dendrimer and further decreased by the coated compound. CSA coating contributed to a reduction in toxicity of more than five times the equivalent amount of dendrimer, in fact the coating was able to mask the free cationic groups of the drug and the peptide dendrimer, reducing interaction with cells and increasing their safety and efficacy. CSA represents one of the components of the placenta extracellular matrix and is described as a receptor for Plasmodium-infected red blood cells (pRBC). CSA conjugation on peptide dendrimer impounded pRBC, reducing in vitro levels of the ring and trophozoite stages of *P. falciparum* [101]. In vivo studies were performed to analyze blood level, tissue distribution, hematological and tissue toxicity of the CQ drug alone, CQ-CSA-coated and CQ-uncoated dendrimers by intravenous administration in albino rats. CSA coating prolonged drug half-life in blood excluding toxicological manifestations [102]. A similar CSA-coated polyethylene glycol-lysine dendrimer was used to encapsulate artemether (ART). Moreover, in this case, conjugation with CSA increased the amount of drug loading and extended the time of drug release. The presence of CSA reduced hemolytic and cellular toxicity. As in the previous study, ART-CSA-coated dendrimers sequestered pRBC, reducing trophozoite stages of *P. falciparum* cultures. Furthermore, in vivo studies showed that CSA coating protracted the permanence time of drugs in the blood [103]. Movellan et al. evaluated the ability of four different dendritic derivatives based on 2,2-bis(hydroxymethyl) propionic acid (bis-MPA) monomers to encapsulate the antimalarial drugs CQ and primachine (PQ). The antimalarial activity of the dendritic derivatives was tested in vitro against *P. falciparum* and in vivo against *Plasmodium yoelii*. The dendritic derivate D showed the lowest cytotoxic effect on the human umbilical vein endothelial cells (HUVEC) with an IC_50_ value of 1.2 mg/mL, reducing the free CQ with an optimal encapsulation efficiency of 100%. The same CQ concentrations in the form of free drugs and complexed drugs were tested on the growth of *P. falciparum*. The findings showed that the D-CQ increased threefold the antiplasmodial activity of free drugs, reducing the IC_50_value from 13.6 nM for free CQ to 4.0 nM for the complexed drug. The in vivo assays in mice did not show a relevant enhancement of free CQ effectiveness. Intraperitoneal administrations of 0.5 and 1 mg/kg for day of free CQ reduced parasitemia of 38.2% and 52.1%, respectively. In contrast, the same amount of drug encapsulated in D produced the respective effects of 34.0% and 43.6% reduction in parasitemia. However, mice survival data showed that D-CQ-treated mice survived for about 9.7 days, while CQ-treatment reduced the survival days to about 7 [101]. Coma-Cros et al. investigated the ability of two micelle carriers based on dendritic macromolecules to target pRBCs, to encapsulate antiplasmodial drugs CQ, PQ and quinacrine (QN), and, lastly, to inhibit the growth of *P. falciparum*. The dendritic derivative used in these studies were: (i) HDLDBC-bGMPA, hybrid dendritic-linear-dendritic block copolymer (HDLDBC) consisting of Pluronic F127 and dendrons derived from 2,2′- bis(glycyl oxymethyl) propionic acid (b-GMPA); and (ii) DHP-bMPA, dendronized hyperbranched polymer (DHP) resulting from 2,2′-bis(hydroxymethyl) propionic acid (bis-MPA) hyperbranched polymers and bis-MPA dendrons complexed with glycine groups. Dendritic derivatives showed no cytotoxic and hemolytic effect up to a concentration of 0.15 mg/mL [104]. Fluorescence microscopy analysis revealed that only DHP-bMPA dendrimers were able to specifically target pRBC, and that their localization was mainly within the plasma membrane of parasites. Furthermore, it was noted that both dendritic derivatives were incorporated into HUVEC cells. In particular, HDLDBC-bGMPA was found within the cytosol, while DHP-bMPA was able to enter the nucleus. These findings suggested the use of these polymers in targeting several kinds of cells and different subcellular sites. CQ, PQ and QN were combined with DHP-bMPA and HDLDBC-bGMPA. DHP-bMPA showed a better encapsulation efficiency, ranging from 37% to 70%, in contrast to HDLDBC-bGMPA, whose loading capacities were between 31% and 48%. All formulations were tested on the growth of *P. falciparum* in vitro. Encapsulated drugs showed IC_50_values resembling those of the free drug, demonstrating an appropriate release of the compounds from dendrimeric derivatives. However, the high specificity of DHP-bMPA for pRBCs reduced unspecific toxicities [101]. In a study from Elmi et al., the activity of chloroquine (CQ)-loaded curcumin-based anionic linear globular dendrimer G2 (NDC-CQ) against *P. falciparum* was evaluated. The water solubility of CQ increased broadly when complexed with dendrimer. The complex exhibited controlled drug-release, in particular, 92% of loaded drugs were released after 48 h. Dendrimers increased the antiplasmodial activity of CQ. The highest cytotoxic effect of NDC-CQ was observed at 2.5 μg/mL where, after 48 h of treatment, 82.6% of the parasites were removed. In contrast, at the same concentration, CQ showed a lower antiparasitic effect (55%). The nanocomposite at a therapeutic dose (2.5 μg/mL) showed negligible toxicity on Vero cell lines and a non-relevant hemolytic effect on human erythrocytes. The metabolic cycles of the parasite after treatment with CQ and NDC-CQ were assessed via NMR and LC-MS methods. The nanocomposite treatments showed their effect on metabolic flavin mononucleotide, L-tyrosine and L-glutamine pathways more than CQ exposure. These pathways are ideal candidates as targets for antimalarial drugs [94].

### 4.2. Leishmaniasis

Leishmaniasis is a neglected protozoan disease caused by the parasite *Leishmania*. Initially, the infection was related to poverty and malnutrition and, as a result, it was diffused within East Africa, South East Asia and Latin America. Nowadays, migrations and travels exacerbated its spread also in developed societies. Estimates report 12 million infected people and 350 million individuals at risk worldwide [105]. In 2017, the WHO reported that the most new cases (94%) were within Brazil, India, Ethiopia, Somalia, Sudan, and Kenya [106]. Deaths due to leishmaniasis are from 20,000 to 40,000 worldwide every year [107], representing the second parasitic disease associated with high mortality after malaria. Unfortunately, the age range mainly affected by the disease is represented by children less than 15 years old [106].

Transmission of leishmaniasis occurs after the bite of female sandfly, an arthropod which populates particularly the Mediterranean countries [108]. Several are the parasite natural reservoirs, i.e., humans, dogs, rodents, wolves and opossums. *Leishmania* clinical diseases are principally three and their outcomes depend on the balance between the species of infective parasite and host’s immune response. There are different forms of leishmaniasis but, among humans, the most common are: visceral, cutaneous and mucocutaneous [109]. Visceral leishmaniasis (VL), also called kala-azar, is due to the infection of the reticuloendothelial system. Parasites infect and replicate into specialized phagocytic cells, invading organs such as liver, spleen and bone marrow. VL is characterized by hepatosplenomegaly and bone marrow suppression [110,111,112,113]. Cutaneous leishmaniasis (CL) is an uncomplicated disease, causing skin lesions [110,114]. In general, they affect the site at which the sandfly bite occurs, for instance the face, neck and limbs. Lesions spontaneously heal, but, in some cases, they can evolve towards mucocutaneous tissue damage. Mucocutaneous leishmaniasis (MCL) is a more severe disease derived from the spread of *Leishmania* from the skin to oral and upper respiratory tract mucosa [115]. MCL outcomes are evident and transfiguring, involving the destruction of nose tissue, mouth, oro- and nasopharynx and eyelids [116]. In addition, the disease can lead to malnutrition and death for occurring secondary infections [117]. The principal chemotherapeutic treatment against leishmaniasis is pentavalent antimony (Sb). Its mechanism of action is not well understood, but it has been reported that Sb blocks the activity of trypanothione reductase (TR) enzyme [118,119,120,121]. Nevertheless, Sb shows high toxicity and is liable to resistance development [122,123]. In order to overcome these unfavorable effects, other anti-leishmanial drugs have been studied, such as amphotericin B (AmB), a polyene-binding ergosterol. AmB antiparasitic activity is mainly due to its ability to induce instability and pores in the parasite membrane [44,124]. Although AmB causes host cell toxicity [125,126] and its use has been associated to several side effects (hemolysis, liver damage, insolubility, fever) [126,127], AmB deoxycholate and liposomal formulations are well tolerated and widely exploited for the treatment of *Leishmaniasis* [128,129,130,131]. Some vaccine candidates have been studied against leishmaniasis. In 2011, a canine vaccine (CaniLeish) was authorized in Europa for dogs [132,133]. CaniLeish efficacy against human leishmaniasis has not yet been proved. Recently, DNA vaccines are gaining considerable importance in providing prevention against *Leishmania* infection [132,134,135].

Daftarian et al. described the use of a novel peptide-dendrimer nanocarrier: it consisted of a Pan-DR-binding-epitope (PADRE) bound to murine and human major histocompatibility complex class II (MHC II) and covalently linked to PAMAM-dendrimer [136]. The resulted complex PADRE-derivatized-dendrimer (PDD) was able to activate antigen presenting cells (APCs), i.e., macrophages and dendritic cells (DC), showing a double action: on the one hand, it carried liposomal AmB (LAmB) at the specific site of infection and, on the other, it induced Th1 response in vivo. In detail, BALB/c mice were infected intraperitoneally with *L. major* promastigotes and then inoculated intravenously with LAmB (37.5 mg/kg/day, the full dose), LAmB (6.25 mg/kg/day low dose), or PDD-LAmB (6.25 mg/kg/day low dose with PDD) once a day for 10 successive days. PDD-LAmB reduces LAmB effective dose and its toxicity. Furthermore, the conjugate increased LAmB efficacy as demonstrated by healing of skin lesions in 100% of PDD-LAmB-treated mice compared to LAmB-treated ones at the full dose. These data were confirmed by a high reduction (86%) in parasite burden in the host. PDD-LAmB also stimulated APCs to elicit a Th1 response against this intracellular pathogen, as indicated by a marked transient lymphadenopathy, and an increase in the MHC II expression and interferon (IFN) γ induction in the PDD-LAmB-treated group. In order to improve AmB targeting towards macrophages and potentiate the immune response, Jain et al. designed another type of dendrimer, namely a multimeric poly(propyleneimine) (PPI) dendrimer conjugated to muramyl dipeptide (MDP) and loaded with AmB, termed MdPPIA [137]. MDP is able to induce macrophage activity in infected mice [138], but the rapid body clearance is a strong limit for its clinical use. The dendrimeric formulation was able to release AmB at higher concentrations, in particular acidic pH conditions mimicking the macrophages’ environment. The toxicity of MdPPIA was reduced both in human erythrocytes and murine macrophages (J774A.1 cells) compared to commercial formulations of AmB, i.e., Fungizone and AmBisome. Furthermore, MdPPIA showed a great potential against VL-infected BALB/c mice reducing the parasite burden of about 90% compared to Fungizone or AmBisome-treated mice (reduction of 46.34% and 61.23%, respectively). This high antileishmanial activity can be explained by the dual action of MdPPIA formulation, acting both in macrophage activation and immune response stimulation, as confirmed by dysregulation of cytokines production. Recently, Mehrizi et al. demonstrated that Anionic Linear Globular Dendrimer (ALGD) was able to incorporate and release AmB reducing its toxicity and improving its solubility [44,139]. Different to the other dendrimers loaded with AmB previously mentioned, ALGD is negatively charged and it has been widely studied as an anticancer agent [140,141,142,143]. It has several advantages mainly related to its simple, economic and green synthesis procedure. ALGD-AmB formulation showed a 484-fold increase in drug solubility and was 100% non-toxic on peritoneal macrophages after 72 h. It killed 70% of *L. major* promastigotes and 63% of amastigotes. Finally, the formulation had a high potential in wound healing and it was able to reduce parasite burden in treated mice compared to the control group (mice treated with AmB alone).

One important feature for the introduction of novel drugs against leishmaniasis is considered the improvement of drug bioavailability and targeted delivery. Therefore a recent study has showed that PEGylated dendritic polyglycerol-based conjugate decorated on the surface with mannose units can be very efficient in the cellular uptake and colocalization with macrophage-residing *Leishmania* parasites [144]. Cellular uptake of mannosylated nanocarriers was quantitatively investigated by flow cytometry and was shown to depend on CD206 receptor by preincubation of macrophages with dextran (a demonstrated competitor of CD206). The study showed that this mannosylated nanoparticle targeted to CD206 receptors could reach a higher accumulation in *Leishmania*-infected macrophages versus non-infected macrophages. Furthermore, the dendrimeric compound with AmB attached via a pH-cleavable hydrazone bond could be useful as a potential weapon against parasites in view of the reduction of the known AmB toxicity.

### 4.3. Schistosomiasis

Schistosomiasis is a devastating helminth infection caused by trematode worms of the genus *Schistosoma*. It is widespread in tropical and subtropical areas affecting as many as 78 countries principally localized in Africa (90%), and the rest in Asia and South America. The WHO estimated that more than 230 million individuals are infected with schistosomes and about 779 million people reside in endemic territories [145]. Three main species are counted in the genus *Schistosoma* and are geographically distributed: (i) *Schistosoma haematobium*, spread in Sub-Saharan Africa and the Nile delta; (ii) *S. japonicum*, limited to China, Indonesia and Philippines; and (iii) *Schistosoma mansoni*, present principally in Sub-Saharan Africa, Brazil and Caribbean islands [146]. People at risk live in poor and rural conditions without the common hygienic-sanitary standards; in addition, migrations and tourism have deeply contributed to the spread of schistosomiasis infections that have reached several novel areas. Young adolescents represent a high-risk population and meanwhile, severity and prevalence of the disease scales down with adulthood. Schistosoma infection occurs through infected water containing intermediate host snails. Here, in the aquatic species, the asexual part of parasite life cycle takes place: it goes on for 4–6 weeks before Schistosoma larval forms are released. These are called cercariae and represent the infective forms for humans. In fact, cercariae are able to penetrate in human skin [147], although some studies reported that the parasitic infection could originate by drinking contaminated water [148]. Humans are the definite host where the parasites realize their sexual reproduction. Firstly, they develop into schistosomula and, after 4–6 weeks in the liver, schistosome production occurs [147]. Adult schistosomes can couple into male–female pairs and move up to mesenteric (*S. haematobium*) or vesical veins (*S. japonicum* and *S. mansoni*). *S. haematobium* impairs urogenital system and can increase the risk of developing bladder cancer [149,150]. In addition, urogenital schistosomiasis is an incremental risk factor for the transmission of Human Immunodeficiency Virus (HIV) and its related AIDS disease progression [151]. *S. japonicum* and *S. mansoni* disseminate into intestine or liver causing anemia, malnutrition [152,153], hepatosplenomegaly [154] and portal hypertension [155]. Rarely, parasitic eggs can spread in ectopic sites as the central nervous system inducing myelopathy, paralysis and neurological impairment [156,157,158]. On the other hand, acute schistosomiasis is very common, especially in travelers and migrants. Typical clinical manifestations are fever, headache, myalgia and fatigue, which persist for 2–10 weeks [159,160]. Treatment of schistosomiasis requires the use of praziquantel (PZQ), which is active against all *Schistosoma* species but, mostly, it removes adult worms and not immature stages. Its mechanism of action has not been completely clarified, although the praziquantel main action is to produce holes in *Schistosoma* tegument, thus unmasking antigens previously hidden to the immune system [161,162,163]. Experimental evidence reported that the drug increased the parasite’s permeability to calcium, resulting in the induction of contraction of the worm’s musculature with consequent paralysis. The damaged parasites are then removed from the host site of infection, brought into the systemic circulation and destroyed through phagocytosis [164]. Due to the emergence of resistance against PZQ and frequent re-infection, schistosomiasis is still not effectively controlled [165]. Artemether and artesunate, well known antimalarial drugs, have been used together with PZQ to eliminate immature *Schistosoma* forms [166,167]. Nevertheless, PZQ can be accompanied by different collateral effects, such as dizziness, headache, abdominal pain and bloody stool [168]. Dendrimers have been exploited by several researchers to pursue goals to eliminate schistosomiasis. Wang et al. designed PAMAM dendrimers conjugated to the *S. japonica* DNA vaccine and evaluated its ability to improve the protective effect against schistosomiasis. The dendrimer 4.0 G PAMAM-Lys were prepared by a Lysine-modified method. The genetic sequence of the 23 kDa membrane protein (SjC23) from *S. japonica* (Chinese strain) was cloned into the eukaryotic expression plasmid, pcDNA3.1. In physiological conditions, PAMAM bound plasmid electrostatically, protecting it from extracellular nucleases. The cytotoxicity of the nanostructure was measured by MTT assay on human kidney transformed cells (293T). The dendrimer showed 80–90% cell viability within the concentration range between 20 to 200 μg/mL. In this study, the transfection efficiency of DNA/PAMAM-Lys and free DNA was assessed. At 48 h after transfection, the cells were harvested and the percentages of transfected cells were measured by fluorescence emission of Enhanced Green Fluorescent Protein (EGFP). The transfection efficiency of DNA/PAMAM-Lys was 75%, while free DNA was barely detected. These findings suggested that PAMAM-Lys was endowed with high cell permeability and transfection ability. The immunogenicity of DNA/PAMAM-Lys and free DNA were assessed by intramuscular administration in mice (100 μg). SjC23-specific antibody responses in mouse sera were evaluated by ELISA. PAMAM-Lys/DNA raised higher levels of anti-SjC23 IgG2a responses compared to only DNA. These results demonstrated the ability of DNA/PAMAM-Lys to induce a specific Th1-type antibody response. Further analysis showed that DNA/PAMAM-Lys induced a massive increase in the production of IL-2 and IFN-gamma. These findings provided a new vector of vaccine administration to increase the immunoreactivity of the DNA vaccine [169].

### 4.4. Toxoplasmosis

Toxoplasmosis is a parasitic infection caused by *Toxoplasma gondii*, an obligate intracellular protozoa, belonging to the phylum Apicomplexa. This parasite is highly diffused in nature worldwide and parasites a consistent range of hosts, including birds and mammals [170]. Toxoplasmosis has a relevant impact on mortality and life quality, in fact, it has been estimated that 8–22% of people are infected in the United States and the United Kingdom. In central America, south America and continental Europe, the prevalence of infection varies from 30% to 90%. Environmental conditions, eating habits, hygiene and host sensitivity can influence the prevalence found worldwide [171]. Toxoplasmosis is transmitted to human hosts from mother to child during pregnancy, by consumption of uncooked infected meat, food or water contaminated with fecal matter, exposure to infected cat feces, blood transfusion or organ transplantation [172]. The life cycle of *T. gondii* consists of a sexual stage within definitive hosts and an asexual stage in intermediate hosts. Definitive hosts belong to the family of Felidae, for instance domestic cats. Intermediate hosts are represented by warm-blooded animals such as cattle and humans [173]. The infection starts when a cat consumes meat contaminated with tissue cysts. The cystic walls collapse due to the presence of digestive enzymes in the stomach. In humans, the clinical signs of toxoplasmosis are mainly observed in immunocompromised individuals and the fetus during pregnancy [174]. Toxoplasmosis can cause important complications in patients with weakened immune systems. *T. gondii* causes encephalitis due to focal intracerebral lesions in immunocompromised individuals [175]. Most healthy people can develop symptoms similar to flu headache, convulsions and tiredness. If untreated, *Toxoplasma* encephalitis can be lethal [176]. During the acute phase of congenital infection, the tachyzoites can overpass the placenta and infect the fetus. The severity of congenital disease depends on the gestation period leading to blindness, mental retardation or spontaneous abortions [172]. Actually, pyrimethamine and sulfadoxine represent the main drugs to treat toxoplasmosis. These drugs block the synthesis of tetrahydrofolic acid, which is essential for the synthesis of nucleic acids of parasites. These drugs are active against tachyzoites but do not eliminate bradyzoites. This limitation, associated with toxic side effects, requires the development of new treatments.

Few studies have evaluated the application of dendrimers in the management of toxoplasmosis [177]. Lai et al. assessed the activity of transductive peptide dendrimers conjugated with phosphorodiamidate morpholino oligomers (PPMO) against *T. gondii*. In vivo studies showed the decrease of live parasites after treatment [178]. In a study conducted by Prieto et al., cationic G4 and anionic G4.5 PAMAM dendrimers, conjugated to sulfadoxine, were tested as anti-toxoplasmic agents. Sulfadoxine, anionic G4.5 PAMAM dendrimers and the complex sulfadoxine-anionic G4.5 PAMAM dendrimers did not display toxicity on Vero and J774 cells after 24 h of exposure. In contrast, cationic G4 PAMAM dendrimers and its complex with sulfadoxine were toxic at concentrations equal or greater than 3.3 μM. The antiparasitic effect of complexed dendrimers was evaluated on Vero cells infected with the *T. gondii* RH strain after 4 h of treatment. Cationic dendrimers showed 60% activity at 0,03 μM, in contrast, anionic dendrimers resulted in a 25% reduction in infection at 33 μM. These results proposed a sulfadoxine-cationic G4 PAMAM dendrimer as a potential drug for *T. gondii* infections [179]. At present, there is no vaccine against toxoplasmosis: therefore, absolute prevention cannot be ensured. Chahal and colleagues developed a novel modified dendrimer nanoparticle (MDNP) vaccine system that protects mice against lethal *T. gondii* infections. MDNP were obtained through a reaction between G1 PAMAM dendrimer and 2-tetradecyl oxirane. They were conjugated to multiple RNA replicons based on the genomes of Venezuelan equine encephalitis virus, which encoded six antigens of *T. gondii* (GRA6, ROP2A, ROP18, SAG1, SAG2A, and AMA1). The chosen antigens appear in several stages of the parasite life cycle and were shared by multiple strains. Mice were treated intramuscularly with a single 40 μg dose of vaccine and exposed to lethal doses of parasite, thirty days after immunization. Animals were monitored for clinical symptoms of the disease. On the 12th day, all immunized mice had survived the infection. This is the first evidence of a replicon RNA-MDNP vaccine system with a single dose able to confer complete protection against *T. gondii* infections [180].

### 4.5. Acanthamebiasis

*Acanthamoebae* is a free-living ameba able to infect humans causing opportunistic infections, that are granulomatous amebic encephalitis (GAE) and amebic keratitis (AK). GAE is a lethal infection of the central nervous system which can also affect the lungs [181]. Generally, the infection occurs together with other diseases in immunocompromised patients, such as systemic lupus erythematosus [182], diabetes [183] and HIV [184,185], but some cases of infection in immunocompetent subjects had also been reported [186,187]. Compared to the huge number of deaths caused by AIDS, GAE lethality is very low: it has been estimated that the approximate rate is 1.57 GAE deaths per 10,000 AIDS deaths in the United States of America (USA) [188]. Nevertheless, GAE remains an important contributing factor in AIDS deaths and its knowledge deserves attention. As previously mentioned, *Acanthamoebae* is also the etiologic agent of AK, a corneal infection which can result in serious eye damage [189,190]. As opposed to GAE, AK occurs in immunocompetent people, in particular in contact lens users (85%) [191,192]. Its incidence had rapidly been growing in the last few years and it has been reported to affect approximately one to two cases per million contact lens wearers in the USA [193]. The lifecycle of *Acanthamoebae* includes two stages: an active trophozoite form and a dormant cyst. Trophozoites are mobile feeding cells are able to eat bacteria, yeast and algae [194,195,196]. Cysts represent the resistant form of *Acanthamoebae*, are immobile and contain a double wall [197]. The resistance encompasses a huge variety of unfavorable conditions, such as desiccation, loss of nutritive sources and temperature/pH switches [198,199]. In addition, cysts do not respond to biocides, antibiotics and they are resistant also to very low temperatures [200,201,202]. Once the environmental setup becomes favorable again, excystment occurs and trophozoites are released [203,204]. AK diagnosis is also problematic: eye infection caused by *Acanthamoebae* is very similar to Herpes Simplex Virus type 2 (HSV-2), *Pseudomonas aeruginosa* and fungal infections [205]. AK diagnosis is generally performed through cytological analysis of corneal scrapes or biopsies, using appropriate staining methods, or through molecular assays (PCR) [205]. The treatment of these infections still represents a major problem due to a lack of sufficiently effective drugs. Amebic keratitis is generally treated with a combination of diamidine (hexamidine or propamidine isethionate) and biguanide (chlorhexidine or polyhexamethylene biguanide-PHMB) while no totally effective treatment for GAE has been found to date. Diamidine and biguanide alter the structure and permeability of the cell membrane, causing cell lysis [206]. These are well-tolerated drugs but prolonged therapy leads to ocular surface toxicity and the development of resistant strains [207]. The resistance to the classical therapeutics and the side effects urgently requires the development of new drugs to efficiently treat *Acanthamoeba* infections. Dendrimers could represent a valid solution to those problems. Heredero-Bermejo et al. evaluated for the first time the activity of cationic dendrimers against *Acanthamoeba castellanii* trophozoites. In particular, the amebicidal potential of three cationic dendrimers was evaluated: 0G-CNN2, 1G-CNN8 and 2G-CNN16 containing one, four and eight generations, respectively, with two ammonium groups by branch were analyzed [208]. The dendrimer efficiency changed according to the generation number and time of exposure. Marked results were obtained with 2G-CNN16. This dendrimer showed amoebicidal activity at LD50 values 0.852 μg/μL after 1 h of exposure, which are consistently lower than the other options. Flow cytometry findings showed that 2G-CNN16 exposure caused a reduction in parasite size and an increase in intracellular granules. Optical and scanning electron microscopy proved that the damage was dose and time-dependent. The same research group studied the activity of several cationic carbosilane dendrimers toward trophozoite of *Acanthamoeba polyphaga*. These nanostructures differed for: (i) nature of core (polyphenoxo “O_3_’’ and silicon atom “Si”); (ii) nature of the fraction close to the ammonium group (sulphur atom “S” and triazole ring “N_3_”); (iii) number of generations (from 1 to 3); (iv) number of terminal groups (4, 6, 8, 11, 12, 16 and 24); and (v) the type of substituents at the ammonium groups (hydrogen “H_3_” or methyl group “Me_3_”). In this study, antiprotozoal potential of 18 dendrimers was evaluated at several concentrations (8–32–128–512 mg/L) and for different exposure times (4–24–48–72 h), as well as their cytotoxicity on HeLa cells. According to the cytotoxicity and trophozoites viability results, only 6 of 18 dendrimers were tested against *Acanthamoeba* trophozoites:G1O_3_(SNH^+^_3_)_6_, G2O_3_(SNMe^+^_3_)_12_, G1Si(SNMe^+^_3_)_4_, G2Si(SNMe^+^_3_)_8_, and G1Si(NMe^+^_3_)_4_. All were not cytotoxic and exhibited a dose and time-dependent effect on the trophozoites. Dendrimers of higher generations showed a similar or greater cytotoxicity compared to amoebicidal activity. Therefore, the selected dendrimers had the lowest generations. Among all of them, the dendrimer G1O_3_(SNH^+^_3_)_6_ showed a comparable effect to chlorhexidine digluconate. IC_50_values of G1O_3_(SNH^+^_3_)_6_ and chlorhexidine digluconate were 2.4 mg/L and 1.63 mg/L, respectively, after 24 h of exposure. The activity of G1O_3_(SNH^+^_3_)_6_ could be due to a minor replacement in ammonium groups, leaving the amines free for interactions. Flow cytometry analysis revealed a reduction in cell size and an increase in the permeability of the cytoplasmic membrane [209].

Martín-Pérez et al. evaluated the action of biguanide-functionalized dendrimers against *A. polyphaga* and *Acanthamoeba griffini.* They exploited two different generations of amine-terminated carbosilane [G1Si(NH_2_)_4_ and G2Si(NH_2_)_8_] to prepare biguanide-functionalized dendrimers (biGU-dendrimers) via microwave chemistry. Amoebicidal activity of the dendrimers was evaluated by direct amoebae counting using a hemocytometer after 24 h of incubation. Biguanide-G2Si(NH_2_)^8^ was the most effective, showing an IC_50_ value of 1.61 and 2.62 μM for *A. griffini* and *A. polyphaga,* respectively. On the other hand, biguanide- G1Si(NH_2_)_4_ was 50% less active, exhibiting IC_50_of 5.58 and 4.61 μM for *A. griffini* and *A. polyphaga,* respectively. The largest number of biguanide groups in G2Si(NH_2_)_8_ (8 groups) could justify the greater efficiency of the higher generation dendrimer in G2Si(NH_2_)_8_ (4 groups). Dendrimers covered with amine groups G2Si(NH_2_)_8_ showed IC_50_ values lower than those obtained for trophozoites. SEM images showed structural damage in treated *Acanthamoeba*. In particular, dimension reduction, loss of form and acanthopodium and cytoplasmic alteration were found [210].

Recent studies used scanning and transmission electron microscopy to analyze the morphological and ultrastructural changes that could be generated on *A. polyphaga* trophozoites and cysts after treatment with cationic dendrimers. The results showed ultrastructural alterations of *A. polyphaga* trophozoites where the cytoplasm was highly disorganized and containing dense granules. The major damage of trophozoites could be attributed to severe alterations in the plasma membrane. Furthermore, the ultrastructure of the cyst also revealed significant changes showing an intracystic shrinkage of the amoeba resulting in a detachment of the plasma membrane from the endocyst. Although the exact mechanism of action is unclear, these results show that membranes are an evident target for the analyzed cationic dendrimers, which are able to interact with the negatively charged plasma membrane. This leads to an increase in cell permeability which can trigger the rupture of the membrane in such a way as to jeopardize the integrity of both trophozoites and cysts. All of these alterations can eventually lead to membrane rupture and cell death [206].

## 5. Conclusions

This review attempted to highlight the potentials of dendrimers as an ideal system for the treatment of parasitic diseases. An in-depth assessment has taken place in recent years and great progress had been made in the field of dendrimers and their application in parasitic diseases. The possibility of designing dendrimers with different properties and functions has led the scientific community to provide their application in the management of parasitic infections. The selective action of dendrimers has been used in the treatment of leishmaniasis and toxoplasmosis to reduce the toxicity associated with amphotericin B. Dendrimers were developed as a carrier of vaccines for the administration of vaccines for the prevention of schistosomiasis infection characterized by the IgG2a antibody response with increased IL-2 production and IFN-γ in vivo. Furthermore, in the treatment of malaria, dendrimers have shown a specific targeting mechanism to plasmodic red blood cells compared to uninfected red blood cells. In light of the literature available from pre-clinical and clinical studies, dendrimers are potential delivery systems for treatment of parasitic diseases; however, further studies are needed to understand their action mechanisms, also considering that the use of dendrimers for combatting parasitic diseases is still in its infancy, and therefore future developments are strongly foreseeable.

## Figures and Tables

**Table 1:** Dendrimer types and their applications.

Type of Dendrimer	Examples	Applications	References
PPI (Poly Propylene Imine) dendrimer	Astramol	Gene delivery and bioimaging	[58,59]
PAMAM (Poly Amido Amine) dendrimer	Tomalia	Anticancer drug delivery, gene delivery and bioimaging	[60,61,62]
Frechet-type dendrimer	Tomalia	Organic synthesis, gene delivery and transfection agent	[63,64]
Core-shell tecto dendrimer	Starburst, Mercapto	Anticancer drug delivery	[65,66]
Chiral dendrimer	Pentaerythritol derivatives	Anticancer drug delivery	[67]
Liquid crystalline dendrimer	Mesogen-functionalized carbosilane	Gene delivery and antimicrobial agent	[68,69]
Peptide dendrimer	Beta Casomorphin (human) derivates	Gene and drug delivery, protein mimics	[32,70]
Multiple antigen peptide dendrimer	VivaGel	Treatment and prevention	[71,72]
Glycodendrimer	Glycodendrimersome	Anticancer drug delivery and vaccine research	[73]
Hybrid dendrimer	Polysilsesquioxanes	Drug delivery	[74]
PAMAMOS (Poly Amidoamine Organosilicon) dendrimer	Silarylene-Siloxane	Engineering and electronics research	[75]
Amphiphilic dendrimer	SuperFect, Bola-amphiphiles	Bioimaging, transfection and antimicrobial agent	[76,77]

## Data Availability

No new data were created or analyzed in this study. Data sharing is not applicable to this article.
